# Comparison of Orbit-Based and Time-Offset-Based Geometric Correction Models for SAR Satellite Imagery Based on Error Simulation

**DOI:** 10.3390/s17010170

**Published:** 2017-01-17

**Authors:** Seunghwan Hong, Yoonjo Choi, Ilsuk Park, Hong-Gyoo Sohn

**Affiliations:** School of Civil and Environmental Engineering, Yonsei University, Seodaemun-gu, Seoul 03722, Korea; hotaeim@yonsei.ac.kr (S.H.); yoonjo15@yonsei.ac.kr (Y.C.); moncher@yonsei.ac.kr (I.P.)

**Keywords:** TerraSAR-X, geolocation error, synthetic aperture radar, error simulation, geometric correction

## Abstract

Geometric correction of SAR satellite imagery is the process to adjust the model parameters that define the relationship between ground and image coordinates. To achieve sub-pixel geolocation accuracy, the adoption of the appropriate geometric correction model and parameters is important. Until now, various geometric correction models have been developed and applied. However, it is still difficult for general users to adopt a suitable geometric correction models having sufficient precision. In this regard, this paper evaluated the orbit-based and time-offset-based models with an error simulation. To evaluate the geometric correction models, Radarsat-1 images that have large errors in satellite orbit information and TerraSAR-X images that have a reportedly high accuracy in satellite orbit and sensor information were utilized. For Radarsat-1 imagery, the geometric correction model based on the satellite position parameters has a better performance than the model based on time-offset parameters. In the case of the TerraSAR-X imagery, two geometric correction models had similar performance and could ensure sub-pixel geolocation accuracy.

## 1. Introduction

The Synthetic Aperture Radar (SAR) satellite system using a microwave signal to observe ground information can collect data over a large area under any weather conditions. With the advantages of a SAR imaging system, SAR images have been used as a powerful solution for various applications such as disaster response [1,2], environmental monitoring [3,4,5], and Digital Elevation Model (DEM) generation [6,7]. In particular, with the development of high-resolution SAR systems such as TerraSAR-X (Germany), KOMPSAT-5 (Korea), COSMO-SkyMed (Italy), and RADARSAT-2 (Canada), the importance of geolocation accuracy, which defines the geometric relationship between ground target and pixel coordinate is highly focused than before [8,9,10,11,12,13,14].

In general, the geometry of the SAR imaging system has been represented by the Range-Doppler model [15,16,17]. In the geometric relationship, the satellite orbit and the sensor information play an important role in geolocation accuracy [11,18,19]. In the past, the accuracy of SAR satellite orbit information was immature [20]. For this reason, geometric correction models to correct satellite position and velocity information had been suggested to achieve sufficient geolocation accuracy of the satellite imagery [21]. Chen and Dowman [22] addressed that the satellite orbit errors have influence on intersection accuracy and proposed the weighted least squares solution for the intersection of stereo SAR images. For the SAR imagery, the time-dependent polynomials can define the orbit model with a high accuracy [23]. With the cubic time-dependent of the satellite position model, a geometric correction can be simplified and sensor velocity can be calculated by the first time derivative of the orbit model [17]. Moreover, Vassilaki and Stamos [24] compared the performance of the cubic polynomials, Lagrange polynomials, Trigonometric interpolation and Chebyshev interpolation based on TerraSAR-X orbit data and suggested that the cubic polynomial can be a practical solution to define the relationship between SAR image space and 3-dimensional object space. 

In case of the TerraSAR-X and TanDEM-X, as the centimeter-level positional accuracy of the satellite orbit information provided by Precise Orbit Determination (POD) technique using a dual-frequency Global Positioning System (GPS) signal can be ensured, the stability of a SAR sensor and the influence by geodynamic effects have been monitored and analyzed in many studies [8,9,10,14,25,26]. Accurate satellite orbit, sensor, and atmospheric condition information can ensure high geolocation accuracy of SAR images [10,25]. However, since it is difficult to obtain accurate information about the SAR imaging system’s parameters and the geodynamic conditions, every single SAR imaging system cannot always guarantee its geolocation accuracy. The geolocation accuracy might be different according to captured time and area due to geodynamic error sources like atmospheric signal delay [18,27]. For this reason, GCP-based geometric correction techniques are still used as an alternative solution for a practical application.

As the geometric correction is performed based on the mathematical model of an observation system and parameterized error sources, the adoption of an unsuitable geometric correction technique or miscalculation of the unnecessary parameters can lead to the deterioration of work-efficiency and data quality. Furthermore, the introduction of an unnecessary parameter can decrease the stability and accuracy of a geometric correction due to the correlation among the parameters [18,28]. Meanwhile, due to the correlation among the parameters, the correlated error sources sometimes can be reduced with a simple geometric correction model. For example, for a small satellite imagery, the polynomial geometric correction model that is based on an orbit model can reduce the correlated error sources such as the time delay in sensor, atmospheric delays, Earth movements, and errors in ephemeris information [17,21,22,23,29].

Furthermore, the theoretical minimum number of GCPs is determined by the number of the parameters calibrated in a geometric correction process. To reduce the uncertainty and improve the precision, a sufficient number of well-distributed and accurate GCPs are usually required. Frequently, acquiring the GCPs of high quality from SAR imagery is difficult on a limited budget or under time constraints and the GCPs of poor quality cause critical errors in refining geometric parameters of the SAR imaging system. Moreover, in emergent situations like a disaster event, it is difficult to achieve accurate GCPs and the inaccurate dataset might have outliers. To alleviate the problems, the geometric correction model with the minimal numbers of GCPs are required [18,22]. When the theoretical number of GCPs is minimized, achieving sufficient geometry of GCPs distribution and detecting outliers might be easier.

Obviously, to achieve accurate and stable geometric correction results, the geolocation error sources of SAR imagery must be considered. However, previous studies on geometric error sources and geometric correction algorithms for SAR satellite imagery have been conducted separately. In this regard, this study compared the so-called orbit-based and time-offset-based geometric correction models with conducting the error simulation of geolocation of the SAR satellite imagery. The influence of satellite position, satellite velocity, drift of spacecraft clock, and signal delay were evaluated as the important information for geolocation accuracy of SAR imagery. For verification of the precision of the suggested algorithms, TerraSAR-X images of which accuracy and stability of orbit and sensor information have been verified [10,14,19,25] and Radarsat-1 images that have the positional error of hundreds of meters in ephemeris information [20,30] were used.

## 2. Error Sources for Geolocation Accuracy

### 2.1. Systematic Effects

To precisely project ground target information on pixels in the SAR image, satellite orbit information play a significantly important role. In the case of Radarsat-1, no longer operational, it had been reported that its orbit ephemeris information had locational errors of hundreds of meters and the geometric correction to correct the orbit model was necessary for the practical application of the produced SAR images [20,31,32]. However, recently provided satellite orbit information can guarantee sub-meter accuracy of sensor position. For instance, TerraSAR-X imagery having sub-meter spatial resolution can guarantee satellite orbit information of 20 cm accuracy [9]. Yoon et al. [14] also analyzed that the satellite position information of TerraSAR-X can be ensured to a 4 cm accuracy. Not only that, Hwang et al. [12] analyzed that the precise orbit determination can provide satellite velocity information having an accuracy of 0.003 cm/s. Unlike an optic satellite imaging system in which sensor orientation information is important, the geolocation accuracy of SAR imagery is affected by the accuracy of satellite velocity information.

When accuracy of the satellite orbit information is ensured, the importance of sensor information such as drift in spacecraft clock and electronic delay increases for accurate pixel allocation in a SAR imaging system [8,16,33,34]. The drift of the spacecraft clock is the time difference between the spacecraft clock and the satellite orbit information [16,34]. The clock bias directly causes an azimuth shift of the pixel coordinates in proportion to the pulse repetition frequency [8,26]. When satellite velocity is very high, the calculated distance between the satellite and the target is distorted and the shift in the range direction occurs due to the clock bias [16]. On the other hand, the electronic delay of the signal is a kind of hardware problem in signal propagation time estimation conducted by radar echo sampling and has a direct effect on measuring the slant range between the SAR sensor and the observed target [16,19]. To measure the traveling time of the transmitted signal, the sensitivity of the timing sensor must be significantly high. Fortunately, in general, the electronic delay is stable and it can be eliminated with periodic monitoring of the satellite system [10,19].

### 2.2. Geodynamic Effects

As well as the systematic information, geodynamic variables have an impact on the geolocation accuracy of SAR imagery. The atmospheric delay has the same influence on the pixel allocation in the range direction like the electronic delay of the signal, and can be divided into tropospheric delay and ionospheric delay [25,35,36]. Frey et al. [11] reported that the zenithal tropospheric delay is 2.3 to 2.6 m at sea level and consists of about a 2.3 m hydrostatic delay and about a 0 to 0.3 m wet path delay in the TerraSAR-X imaging system. The effect of the liquid delay is very small and can be negligible in the SAR imaging system [36,37]. In contrast, the ionospheric delay is proportional to the Total Electron Content (TEC) of the atmosphere and the inverse square of the carrier frequency of the signal [38,39]. For the signal where the frequency is higher than the L-band, the tropospheric delay has a larger effect on the path delay than does the ionospheric delay [36,39]. As the general SAR satellite system uses X-band, the influence of the ionospheric delay is relatively small [27,40,41]. In case of the C-band and L-band, the ionosphere causes about 1.5 m range delay and about 25 m range delay, respectively [42]. To achieve a sub-meter geolocation accuracy of a SAR imaging system, the ionospheric delay should be removed based on in-situ observation of atmospheric conditions [10,25].

Solid Earth Tides, caused by the lunar and solar tidal forces acting on the Earth, also has a slight impact on geolocation accuracy. Generally, Earth motions in the vertical direction is larger than that in an eastward or northward direction, and vertical displacements of up to half a meter can occur at the equator [43,44]. Furthermore, ocean tide loading causes Earth deformation, and a millimeter level of periodical fluctuation typically occurs inland [45]. The geolocation error caused by Earth’s motion is so small and generally neglected.

As previous researches verified, in case of the TerraSAR-X imaging system, accurate information of the satellite orbit, sensor timing, and atmospheric condition can ensure geolocation accuracy at the sub-meter level [8,14,19,27]. Furthermore, if the Earth’s motion is precisely modeled with the accurate information of the systematic and geodynamic conditions, centimeter-level geolocation accuracy can be achieved [10,25]. However, it is difficult to obtain a real-time observation data of the error sources over a large area during the image-capturing period. For this reason, GCP-based geometric corrections to adjust the SAR imaging parameters have been applied for a practical SAR imagery application. 

## 3. Geometric Correction Model

Geometric correction is the process to estimate error sources and characterize them in terms of the appropriate parameters [15]. To constructing the geometric correction model of satellite SAR imaging system, the geometric relationship between pixel location and the ground target location needs to be defined. From the defined geometric model, the target parameters are determined to remove the geometric errors resided in the satellite orbit, the stability of sensor, and the atmospheric condition.

Assuming that the processing parameters related to Doppler frequency, slant range, and satellite orbit information are given, the SAR imaging geometry can be physically defined by the Range and Doppler equations [15,18]. The Range and Doppler equations can be represented by Equations (1) and (2):

(1) $$R(t)=|{\vec{P}}^{s}(t)-{\vec{P}}^{T}(t)|$$

(2) $$f_{DC}(t)=\frac{2({\vec{V}}^{s}(t)-{\vec{V}}^{T}(t))\times ({\vec{P}}^{s}(t)-{\vec{P}}^{T}(t))}{\lambda |{\vec{P}}^{s}(t)-{\vec{P}}^{T}(t)|}$$

where, R(t) is the range distance, $f_{DC}(t)$ is the Doppler value which is the difference between Doppler centroid and Doppler shift, t is pixel sampling timing, ${\vec{P}}^{s}$ and ${\vec{V}}^{s}$ are the sensor position and the velocity, ${\vec{P}}^{T}$ and ${\vec{V}}^{T}$ are the target point position and the velocity on the ground, and λ is the radar wavelength. The Range and Doppler equations are solved simultaneously to calculate the image coordinates corresponding to the ground coordinates.

Basically, the Range and Doppler equations contain satellite position, velocity, and sampling timing. The satellite position and the velocity are varying during image acquisition and thus time-dependent polynomial equations can be applied to the satellite orbit model [2,17,18,46]. The satellite position is defined as a quadratic time-dependent equation. The satellite velocity vector is defined as the time-differential of the satellite position vector to minimize the number of required GCPs for geometric correction. The equations of the satellite position and the velocity are represented by Equations (3) and (4):

(3) $${\vec{P}}^{s}(t)=\left[\begin{array}{c} X^{s}(t)\\ Y^{s}(t)\\ Z^{s}(t) \end{array}\right]=\left[\begin{array}{c} a_{0}+a_{1}t+\frac{1}{2}a_{2}t^{2}\\ b_{0}+b_{1}t+\frac{1}{2}b_{2}t^{2}\\ c_{0}+c_{1}t+\frac{1}{2}c_{2}t^{2} \end{array}\right]$$

(4) $${\vec{V}}^{s}(t)=\left[\begin{array}{c} V_{X}^{s}(t)\\ V_{Y}^{s}(t)\\ V_{Z}^{s}(t) \end{array}\right]=\left[\begin{array}{c} a_{1}+a_{2}t\\ b_{1}+b_{2}t\\ c_{1}+c_{2}t \end{array}\right]$$

where, ${\vec{P}}^{s}(t)$ is satellite position vector, ${\vec{V}}^{s}(t)$ is satellite velocity vector, and $a_{0},a_{1},a_{2},b_{0},b_{1},b_{2},c_{0},c_{1},c_{2}$ are satellite position parameters. Generally, header files of SAR images provides ephemeris information to calculate the satellite position parameters [9,32]. 

The pixel sampling timing can be determined by Newton-Raphson methods based on the Doppler equation. By Equation (5), the pixel sampling timing is calculated iteratively until it is converged:

(5) $$t_{new}=t_{old}-\frac{f_{DC}(t_{old})}{f_{DC}^{\prime }(t_{old})}$$

Using the sampling timing, pixel coordinates in the azimuth direction (i(t)) is calculated by the Equation (6):

(6) $$i(t)=\frac{t-t_{0}}{1/PRF}$$

where, $t_{0}$ is offset in the azimuth direction that includes the drift in the spacecraft clock, and PRF is pulse repetition frequency that determines pixel spacing in the azimuth direction. Note that $t_{0}$ might have an influence on the satellite position, velocity information, and the geometric quality of a satellite SAR image. 

For the pixel allocation in range direction, TerraSAR-X and Radarsat-1 imaging systems use different models. In the paper, the used TerraSAR images were available as a slant range imagery but the used Radarsat-1 images were provided by ground range images with polynomial coefficients to transform slant range to ground range [32].

In the case of TerraSAR-X imagery, pixel coordinates in the range direction (j(t)) can be determined by the Equation (7):

(7) $$j(t)=\frac{R(t)-R_{0}}{dP_{range}}$$

where, $R_{0}$ are offset in the range direction including the ranging offset derived by electronic and atmospheric delay of signal, and $dP_{range}$ is projected pixel spacing in the range direction. Note that $R_{0}$ is the parameter of a distance having a linear relationship with the time delay of signal.

In contrast, in case of Radarsat-1 imagery, the slant range (R(t)) between the SAR sensor and the target is converted to ground range ($R_{g}(t)$) based on a 5th degree polynomial equation provided by the image header file [32]. The equation can be represented by the Equation (8):

(8) $$R(t)=\sum_{k=0}^{k=5}(g_{k}\times R_{g}(t)),j(t)=\frac{R_{g}(t)}{dP_{range}}$$

where $g_{k}$ are coefficients provided by the image header file, and j(t) is pixel coordinate in the range direction. However, since the 5th polynomial calculation requires a heavy calculation time, a reduced model can be used to calculate the pixel coordinate in the range direction [46]. The model can be represented by the Equation (9):

(9) $$j(t)=\sum_{k=0}^{k=5}s_{k}{\left(R(t)-R_{0}\right)}^{k}$$

where, $s_{k}$ represents the coefficients for the reduced model.

In the defined geometric models, the error sources in the orbit information of a satellite platform, the time delay of a SAR sensor, and the atmospheric delay are parameterized. When defining a geometric correction model, the adoption of appreciate error parameters is important. To achieve sufficient accuracy, suitable parameters must be adopted in the geometric correction model. Moreover, for stability and efficiency of the geometric correction, the correlation among corrected parameters and minimizing a number of required GCPs must be considered. In the past, satellite orbit information were generally adopted for the geometric correction of a SAR imagery due to an immature accuracy of the ephemeris information [20,29,47,48,49]. However, recent SAR satellite systems such as TerraSAR-X and KOMPSAT-5 have estimated satellite position information of a centimeter-level accuracy by precise orbit determination based on dual-frequency GPS signal [12,14]. By ensuring the accuracy of the orbit information, the drift in a spacecraft clock and the signal delay have been focused to improve the geolocation accuracy of a SAR imaging system [8,16,33,34]. Real-time observation and continuous monitoring of the error sources can reduce the error sources of geolocation but it is difficult for general use of a satellite SAR imagery [19]. For this reason, the GCP-based geometric correction is still widely utilized as a practical solution to achieve sub-pixel geolocation accuracy. 

Even though there are various geometric correction models, the correction models did not reflect the pattern of the geometric distortion caused by geolocation error sources. In this paper, two geometric correction models were applied on the experiments using TerraSAR-X and Radarsat-1, and their performance was evaluated with simulation tests for the error sources in satellite orbit information and sensor timing information. The main parameters of the geometric correction models were determined from the pixel allocation model of the satellite SAR satellite system. The designed geometric correction models are summarized as below:

1. The geometric correction model based on the orbit information errors parameterized by $a_{0},a_{1},a_{2},b_{0},b_{1},b_{2},c_{0},c_{1},c_{2}$ of the Equation (3).
2. The geometric correction model based on the time-offset parameters: the drift in the spacecraft clock and the signal delay parameterized by $t_{0}$ of the Equation (6), and $R_{0}$ of Equations (7) and (8), respectively.

The GCP-based geometric correction models of SAR imagery can be mathematically defined by the least squares solution to minimize the geometric inconsistency that defines the relationship between an actual observation and the parameters of the satellite SAR imaging model [18,28,50]. Moreover, to deal with the nonlinear equations in the SAR imaging system, the first-order Taylor series approximation is applied and the calibration parameters are corrected iteratively until the solution is converged. The linearized observation equations can be written as the matrix form represented by the Equation (10) [50]:

(10) $$\underset{n\times 1}{w}=\underset{n\times m}{J}\;\underset{m\times 1}{\Delta \xi }+\underset{n\times 1}{e}c$$

where, w is a misclosure vector between observed and estimated values, J is Jacobian matrix that consists of the partial derivatives of the Range and Doppler equations taken with respect to the calibration parameters to correct, Δξ is correction vector of corrected parameters, e is an error vector, n is the number of observations, and m is the number of corrected parameters. 

Between the two geometric correction models for the satellite orbit and the sensor information, the Jacobian matrices have difference in accordance with the corrected parameters. The Jacobian matrix in the adjustment model to correct the nine satellite position parameters ($a_{0},a_{1},...,c_{2}$), and can be calculated by the Equation (11):

(11) $$J=\left[\begin{array}{cccc} \frac{\partial f_{DC}(t_{1})}{\partial a_{0}} & \frac{\partial f_{DC}(t_{1})}{\partial a_{1}} & \ldots & \frac{\partial f_{DC}(t_{1})}{\partial c_{2}}\\ \frac{\partial R(t_{1})}{\partial a_{0}} & \frac{\partial R(t_{1})}{\partial a_{1}} & \ldots & \frac{\partial R(t_{1})}{\partial c_{2}}\\ \vdots & \vdots & \vdots & \vdots \\ \frac{\partial R(t_{n})}{\partial a_{0}} & \frac{\partial R(t_{n})}{\partial a_{1}} & \ldots & \frac{\partial R(t_{n})}{\partial c_{2}} \end{array}\right]$$

where, $t_{1},t_{2},...,t_{n}$ are sampling timing of GCPs for geometric correction. 

On the other hand, the Jacobian matrix in the adjustment model to correct the spacecraft clock drift ($t_{0}$) and the signal delay ($R_{0}$) can be written by the Equation (12):

(12) $$J=\left[\begin{array}{c} \frac{\partial f_{DC}(t_{1})}{\partial t_{0}}\\ \frac{\partial R(t_{1})}{\partial t_{0}}\\ \vdots \\ \frac{\partial R(t_{n})}{\partial t_{0}} \end{array}\begin{array}{c} \frac{\partial f_{DC}(t_{1})}{\partial R_{0}}\\ \frac{\partial R(t_{1})}{\partial R_{0}}\\ \vdots \\ \frac{\partial R(t_{n})}{\partial R_{0}} \end{array}\right]$$

As for the intermediate least squares solution, $\Delta \hat{\xi }$ can be calculated by the Equation (13):

(13) $$\Delta \hat{\xi }={\left(J^{T}PJ\right)}^{-1}J^{T}Pv$$

Then, $\Delta \hat{\xi }$ is added to the previous parameter ($\xi_{0}$). The process is repeated until $\Delta \hat{\xi }$ becomes sufficiently small.

In the geometric correction process, the number of GCPs must be considered with the number of the corrected parameters. Unless the number of observations in the least squares solution is sufficient with comparison to the number of the corrected parameters, the system will obviously be deficient [28]. As the pixel coordinate of a SAR imagery is determined by the Range and Doppler equations, one GCP represents two observation equations. The two geometric correction models to correct the satellite orbit parameters and to correct the time-offset parameters are designed to correct nine parameters ($a_{0},a_{1},a_{2},b_{0},b_{1},b_{2},c_{0},c_{1},c_{2}$) and two parameters ($t_{0}$, $R_{0}$). For this reason, at least five GCPs and one GCP are theoretically required to apply the two geometric correction models, respectively.

## 4. Experiments and Results

### 4.1. Datasets

Three TerraSAR-X images in SpotLight mode and two Radarsat-1 images in Fine Mode were used for the error simulation and the evaluation of the geometric correction models. Table 1 and Table 2 summarize the image information used for the experiments. Since the TerraSAR-X images of 4 April 2009 and 23 July 2009 were captured in the same imaging mode, orbit direction, look direction, and incidence angle, the projected spacing and the image size of both images were almost identical. Alternatively, projected spacing and image size of the TerraSAR-X image of 9 April 2009 was different from the other images due to different incidence angle. Since both Radarsar-1 images of 3 August 2005 and 27 August 2005 were captured in similar geometry, the projected spacing and image size of them were similar. 

**Figure 1:**
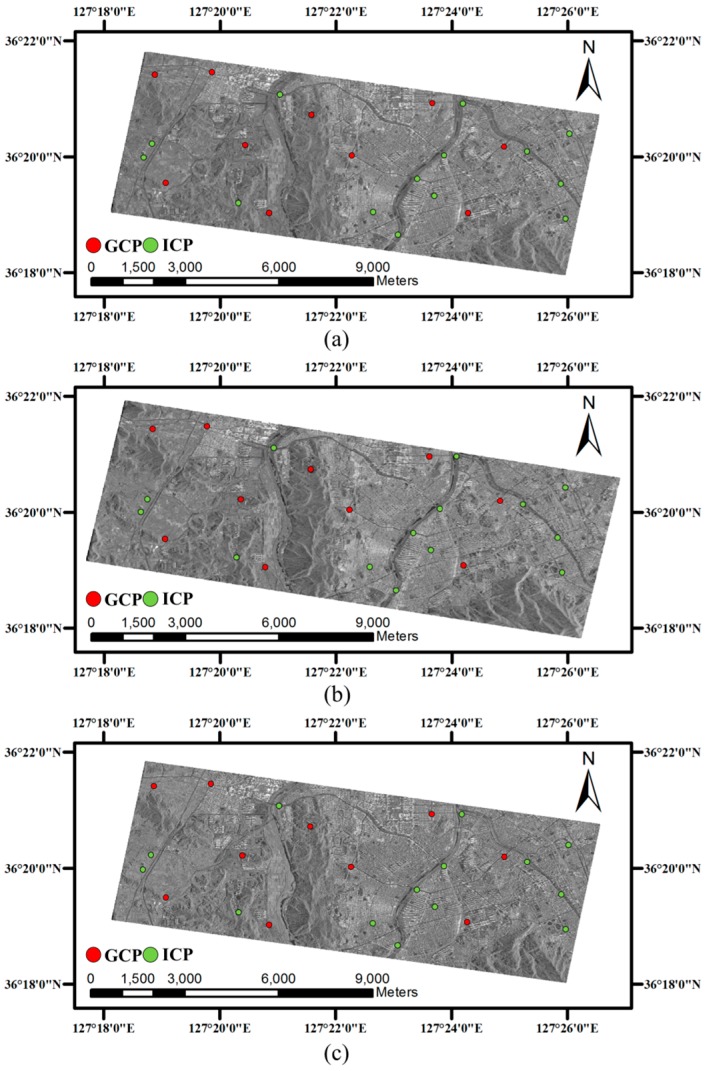
TerraSAR-X images and location of GCPs and ICPs: (**a**) TSX 1 (4 April 2009); (**b**) TSX 2 (9 April 2009); (**c**) TSX 3 (23 July 2009).

**Figure 2:**
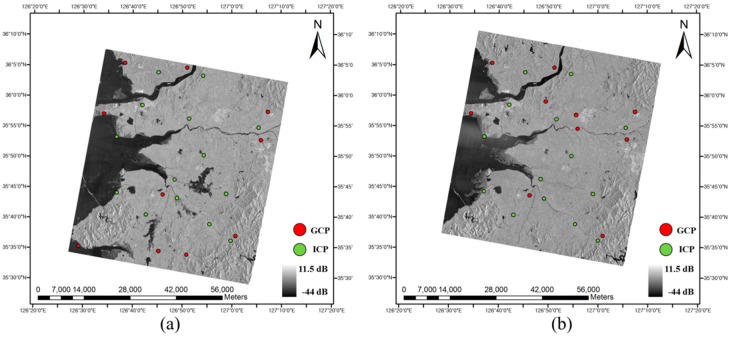
Radarsat-1 images and location of GCPs and ICPs: (**a**) RSAT 1 (3 August 2005); (**b**) RSAT 2 (27 August 2005).

To analyze geometric error budget simulation and to apply the geometric models for SAR images, it was necessary to extract sufficient GCPs of which image coordinates and ground coordinates were known. From each SAR image, image coordinates of the control points were matched with aerial orthorectified images of 50 cm spatial resolution and elevation information of the control points were extracted from a 1:5000 digital topographic map provided by the Korean National Geographic Information Institute [51]. Ten control points were used as GCPs for geometric correction and others were used as Independent Check Points (ICPs) to verify the accuracy of the geometric correction. Locations of GCPs and ICPs are described in Figure 1 and Figure 2.

As shown in Figure 1 and Figure 2, TerraSAR-X images and Radarsat-1 images observed Daejeon, Korea; located 36°20′ N and 127°22′ E, and Jeollabuk-do, Korea; located 36°50′ N and 126°50′ E. Both test sites include various land covers such as urban area, mountainous area, agricultural land, grassland, bare land, wetland, and water area. The elevation range of Daejeon and Jeollabuk-do were 45 m to 457 m and 0 m to 605 m, respectively.

### 4.2. Geometric Correction Results

**Figure 3:**
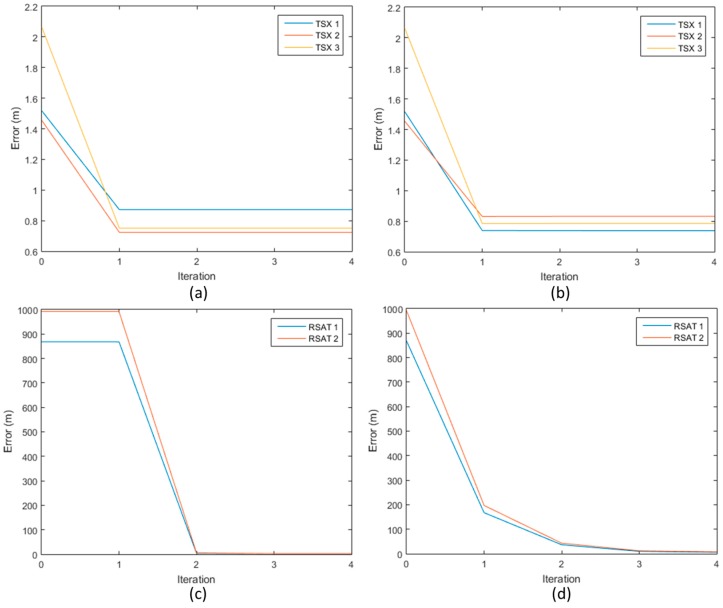
Convergence of parameters in iterative geometric correction process: (**a**) geometric correction of TerraSAR-X based on orbit parameters; (**b**) geometric correction of TerraSAR-X based on time-offset parameters; (**c**) geometric correction of Radarsat-1 based on orbit parameters; (**d**) geometric correction of Radarsat-1 based on time-offset parameters.

Based on the defined geometric model for the pixel allocation and the header information of the TerraSAR-X and Radarsat-1 images, the experiments to evaluate the performance of each geometric correction models were conducted. The geometric correction was conducted using GCPs and the geolocation accuracy of the SAR imaging system before and after the geometric correction were calculated using ICPs as reference points. By iteratively minimizing the inconsistency of the geometric relationship between 3-D ground coordinate and 2-D SAR image coordinates, the parameters representing error sources could be corrected. Figure 3 describes the convergence of the corrected parameters in iterative geometric correction process.

As shown in Figure 3, the parameters could be stably converged within five times of iterations. Before the geometric correction, while the TerraSAR-X images had high geolocation accuracy, the Radarsat-1 images, which had an inaccurate satellite orbit information had low geolocation accuracy. However, in every cases, the iterative parameter optimization process could be successfully converged. The geolocation accuracy before and after the geometric correction are summarized in Table 3. 

In the case of TerraSAR-X, the pixel allocation accuracy using the original geometric model was 1.43 m to 1.95 m. The errors in the range direction were larger than those in the azimuth direction. This is because there was an atmospheric delay in the range direction. After applying two geometric correction models, a 0.72 m to 0.87 m of accuracy could be achieved. For the RMS residuals in the calibration model using GCPs, the model based on orbit information had a better precision than the model based on the time-offset information. This is because the model of the satellite position could offset the effect of the azimuth and the range shift. However, as shown in Table 3, there was no significant difference between the RMS errors in the two models. Even after applying the calibration models, the bias in the range direction was larger than the bias in the azimuth direction. This is because the elevation value of GCPs, which were extracted from the 1:5000 topographic map, might not be accurate enough to offset more shift in the range direction. Since the identification of the accurate coordinates of GCPs is a difficult task, using a larger number of GCPs than the theoretical minimum can reduce the uncertainty of GCP accuracy and improve the precision of the geometric correction.

In comparison, the results of the geometric correction for the Radarsat-1 images of which significantly large errors in the ephemeris information showed significant improvement in the geolocation accuracy. Before applying the geometric correction, the locational errors of the images were 870.57 m and 995.86 m, respectively. However, the images with refined models could ensure locational accuracy of about 1 pixel. Although the model based on the orbit information had better precision, the geometric correction based on the time-offset information could achieve about a 1 pixel level of accuracy. The result implies that the errors in the satellite orbit were not too large to cause significant geometric distortion of the SAR images.

## 5. Error Simulation and Discussion

Geolocation accuracy is obviously affected by the accuracy of satellite orbit and time-offset parameters, and the geometric correction model based on each error factor could be a practical solution. In particular, even though significantly low accuracy of the satellite orbit information of the Radarsat-1 image caused the geolocation error of hundreds of meters, 1 pixel accuracy could be achieved by the geometric correction based on the time drift in a spacecraft clock and the signal delay. In the paper, the performance of the geometric correction models is analyzed based on the error simulation of the satellite orbit information, time drift, and signal delay using the 24 control points extracted from each image (Figure 1 and Figure 2). For the simulation test, the biases for satellite orbit, spacecraft clock, and signal delay were added to the information derived from the header files of the SAR images. The error simulation results are summarized in Table 4.

As shown in Table 4, in the case of TerraSAR-X, a simulated 700 m satellite positioning bias was applied in each direction, and the mean values and the standard deviations of pixel allocation error occurred of up to 627.794 m and 2.998 m, respectively. Moreover, in the case of Radarsat-1, the 700 m satellite positioning bias in each direction caused a pixel allocation error of which the mean values and standard deviations were up to 1020.430 m and 14.866 m, respectively. In contrast, with 0.02 m satellite position biases, the mean values of simulated errors were up to 0.160 m and the standard deviations were below 1 cm. Furthermore, the mean values of the simulated errors caused by 0.001 m/s satellite velocity biases were up to 0.119 m. In contrast, with 0.02 m satellite position biases, the mean values of simulated errors were up to 0.160 m and the standard deviations were below 1 cm. The result implies that the accuracy of the satellite orbit information has an important role for geolocation accuracy and the orbit information determined with dual-frequency GPS signals can ensure a sufficient accuracy for the general application of SAR imagery. 

Moreover, the standard deviations derived by the satellite orbit information errors mean that large errors in satellite orbit information might cause geometric distortion for the geolocation accuracy of a SAR image. On the other hand, the standard deviations of the errors simulated with the drift in the spacecraft clock and the signal delay were zero. Furthermore, the errors of the drift in spacecraft clock and signal delay are linearly correlated with geolocation errors in the azimuth and range direction, respectively. Accordingly, as the small errors in the satellite orbit information caused a shift in the pixel coordinates in the azimuth and range directions, the correction model for the clock drift and signal delay could correct the geolocation errors for TerraSAR-X images having high accuracy of satellite orbit information. However, if the satellite position information has an error of hundreds of meters, geometric distortion might occur and a correction model based on time-offset parameters might not ensure sub-pixel accuracy. When the expected accuracy of the satellite orbit information is too low and a sufficient number of GCPs can be achieved, the geometric correction model based on satellite orbit parameters is recommended.

## 6. Conclusions

Geolocation accuracy is one of the most important factors for the application of SAR imagery. Geometric correction is the process to release the influence of unpredictable errors in SAR sensor information and geodynamic variables such as atmospheric condition by characterizing error patterns in terms of suitable parameters. To achieve sub-pixel geolocation accuracy, a suitable correction model to adjust the geometric relationship between the image and the ground coordinate must be adopted. In this paper, two geometric correction models to adjust the satellite position parameters and the time-offset parameters were evaluated using TerraSAR-X images of which a high accuracy of the satellite orbit and the sensor information have been reported in existing researches and Radarsat-1 images which have large errors in the satellite orbit information.

For Radarsat-1 and TerraSAR-X images, two geometric correction models could significantly reduce geolocation errors caused by errors in the satellite orbit and the sensor information, and the geodynamic variables, and they showed remarkable performance, even though Radarsat-1 images had locational errors of several hundred meters. The results implied that the actual geometric distortion of Radarsat-1 imagery caused by orbit information error was not significantly high. However, since the model based on time-offset parameters cannot reduce the geometric distortion if the errors of the satellite position and velocity are too large, the time-offset-based geometric correction method should be utilized carefully. In this regard, if there are sufficient GCPs in the captured SAR imagery, the geometric correction based on the satellite orbit parameters could be a practical solution to achieve sub-pixel geolocation accuracy. Alternatively, when the accuracy of the orbit information can be guaranteed or the geometric correction is conducted with a limited number of GCPs in emergent situation, the geometric correction based on the time-offset parameters is recommended.

## Figures and Tables

**Table 1:** TerraSAR-X (TSX) image information.

	TSX 1	TSX 2	TSX 3
Imaging Mode	SpotLight	SpotLight	SpotLight
Orbit direction	Descending	Descending	Descending
Look Direction	Right	Right	Right
Acquisition Date	4 April 2009	9 April 2009	23 July 2009
Incidence angle	39.63°	22.25°	39.63°
Wavelength	0.031 m (X-band)	0.031 m (X-band)	0.031 m (X-band)
PRF	8201.417 Hz	8201.419 Hz	8201.391 Hz
Projected spacing (Azimuth/Range)	0.86 m × 0.91 m	0.86 m × 0.45 m	0.86 m × 0.91 m
Image size (Azimuth/Range)	6041 × 8352	6010 × 10,848	5988 × 8352

**Table 2:** Radarsat-1 (RSAT) image information.

	RSAT 1	RSAT 2
Imaging Mode	Fine	Fine
Orbit direction	Descending	Descending
Look Direction	Right	Right
Acquisition Date	3 August 2005	27 August 2005
Incidence angle	38.97°	38.96°
Wavelength	0.056 m (C-band)	0.056 m (C-band)
PRF	1291.340 Hz	1291.031 Hz
Projected spacing (Azimuth/Range)	6.25 m × 6.25 m	6.25 m × 6.25 m
Image size (Azimuth/Range)	10,006 × 9036	10,008 × 9039

**Table 3:** Results of geometric correction based on orbit parameters and time-offset parameters.

SAR Imagery	Before Geometric Correction (m)		After Geometric Correction Based on Orbit Parameters (m)			After Geometric Correction Based on Time-Offset Parameters (m)		
	ICP Errors (Azimuth, Range)	ICP RMS Errors	GCP RMS Residuals	ICP Errors (Azimuth, Range)	ICP RMS Errors	GCP RMS Residuals	ICP Errors (Azimuth, Range)	ICP RMS Errors
TSX 1	−0.47, 1.19	1.43	0.29	−0.06, 0.41	0.87	0.56	−0.04, 0.12	0.74
TSX 2	−0.83, 0.98	1.48	0.42	0.16, 0.04	0.72	0.63	0.06, −0.21	0.83
TSX 3	−0.59, 1.73	1.95	0.32	−0.04, 0.56	0.75	0.49	−0.05, 0.18	0.78
RSAT 1	778.60, −389.43	870.57	2.36	0.97, −0.32	3.67	5.17	1.51, 1.39	4.90
RSAT 2	917.24, −387.80	995.86	2.18	1.50, −1.90	4.34	6.86	2.71, 1.18	6.73

**Table 4:** Results of geolocation error simulation of orbit information and time-offset information.

Error Source		Simulation Results (m) (Azimuth/Range)					
			TSX 1	TSX 2	TSX 3	RSAT 1	RSAT 2
Satellite position error (m)	(X) 700	Mean	−150.659/627.794	−132.610/509.963	−150.672/627.804	141.608/1018.128	141.710/1020.430
		Std. Dev.	0.096/1.011	0.091/2.534	0.096/1.010	1.350/13.682	1.128/13.594
	(Y) 700	Mean	389.985/−111.657	379.755/−264.306	387.989/−111.623	−396.259/−209.717	−396.269/−211.913
		Std. Dev.	0.092/2.399	0.093/2.998	0.092/2.399	1.241/14.866	1.058/14.856
	(Z) 700	Mean	−545.411/−252.908	−521.558/−322.164	−545.425/−252.898	559.249/−406.092	559.068/−408.538
		Std. Dev.	0.106/1.457	0.100/1.564	0.106/1.457	1.180/13.884	0.943/13.349
	(X) 0.02	Mean	−0.043/0.179	−0.038/0.146	−0.043/0.179	0.040/0.291	0.040/0.291
		Std. Dev.	0/0	0/0.001	0/0	0/0.004	0/0.004
	(Y) 0.02	Mean	0.111/−0.032	0.109/−0.076	0.111/−0.032	−0.113/−0.060	−0.113/−0.060
		Std. Dev.	0/0.001	0/0.001	0/0.001	0/0.004	0/0.004
	(Z) 0.02	Mean	−0.156/−0.072	−0.149/−0.092	−0.156/−0.072	0.160/−0.116	0.160/−0.117
		Std. Dev.	0/0	0/0.001	0/0	0/0.004	0/0.004
Satellite velocity error (m/s)	(X) 5	Mean	−378.500/0.120	−260.658/0.070	−378.518/0.120	596.968/−0.326	595.457/−0.327
		Std. Dev.	1.997/0	1.996/0	1.997/0	8.947/0.001	8.733/0.001
	(Y) 5	Mean	67.491/0.004	135.302/0.019	67.473/0.004	−122.646/−0.014	−123.347/−0.014
		Std. Dev.	1.201/0	1.172/0	1.201/0	5.295/0.002	5.293/0.002
	(Z) 5	Mean	152.463/0.019	164.662/0.028	152.461/0.020	−238.238/−0.052	−238.541/−0.053
		Std. Dev.	0.324/0	0.361/0	0.324/0	1.920/0.002	1.575/0.002
	(X) 0.001	Mean	−0.076/0	−0.052/0	−0.076/0	0.119/0	0.119/0
		Std. Dev.	0/0	0/0	0/0	0/0	0/0
	(Y) 0.001	Mean	0.014/0	0.027/0	0.014/0	−0.024/0	−0.025/0
		Std. Dev.	0/0	0/0	0/0	0/0	0/0
	(Z) 0.001	Mean	0.031/0	0.033/0	0.031/0	−0.048/0	−0.048/0
		Std. Dev.	0/0	0/0	0/0	0/0	0/0
Drift in spacecraft clock (100 μs)		Mean	−0.693/0	−0.668/0	-0.693/0	0.664/0	0.664/0
		Std. Dev.	0/0	0/0	0/0	0/0	0/0
Signal delay (10 ns)		Mean	0/−1.469	0/−1.410	0/−1.469	0/−2.389	0/−2.396
		Std. Dev.	0/0	0/0	0/0	0/0	0/0

## References

[1] Song Y.-S., Sohn H.-G., Park C.-H. (2007). Efficient water area classification using radarsat-1 SAR imagery in a high relief mountainous environment. Photogramm. Eng. Remote Sens..

[2] Hong S., Jang H., Kim N., Sohn H.-G. (2015). Water area extraction using radarsat SAR imagery combined with landsat imagery and terrain information. Sensors.

[3] Akbari V., Doulgeris A.P., Eltoft T. (2014). Monitoring glacier changes using multitemporal multipolarization SAR images. IEEE Trans. Geosci. Remote Sens..

[4] Kim J.-W., Lu Z., Jones J.W., Shum C., Lee H., Jia Y. (2014). Monitoring everglades freshwater marsh water level using L-band synthetic aperture radar backscatter. Remote Sens. Environ..

[5] Yuan T., Lee H., Jung H.C. (2015). Toward estimating wetland water level changes based on hydrological sensitivity analysis of palsar backscattering coefficients over different vegetation fields. Remote Sens..

[6] Toutin T., Gray L. (2000). State-of-the-art of elevation extraction from satellite SAR data. ISPRS J. Photogramm. Remote Sens..

[7] Crosetto M. (2002). Calibration and validation of SAR interferometry for dem generation. ISPRS J. Photogramm. Remote Sens..

[8] Breit H., Fritz T., Balss U., Lachaise M., Niedermeier A., Vonavka M. (2010). Terrasar-x SAR processing and products. IEEE Trans. Geosci. Remote Sens..

[9] DLR Terrasar-x: Ground Segment: Basic Product Specification Document (tx-gs-dd-3302). http://www.dlr.de/PortalData/1/Resources/raumfahrt/weltraum/TX-GS-DD-3302_Basic-Product-Specification-Document_1.5.pdf.

[10] Eineder M., Minet C., Steigenberger P., Cong X., Fritz T. (2011). Imaging geodesy—Toward centimeter-level ranging accuracy with terrasar-x. IEEE Trans. Geosci. Remote Sens..

[11] Frey O., Meier E., Nüesch D., Roth A. (2004). Geometric error budget analysis for terrasar-x. Proceedings of the 5th European Conference on Synthetic Aperture Radar EUSAR, 2004.

[12] Hwang Y., Lee B.-S., Kim Y.-R., Roh K.-M., Jung O.-C., Kim H. (2011). Gps-based orbit determination for kompsat-5 satellite. ETRI J..

[13] Yoon J., Keum J., Shin J., Kim J., Lee S., Bauleo A., Farina C., Germani C., Mappini M., Venturini R. Kompsat-5 SAR design and performance. Proceedings of the 2011 3rd International Asia-Pacific Conference on Synthetic Aperture Radar (APSAR).

[14] Yoon Y.T., Eineder M., Yague-Martinez N., Montenbruck O. (2009). Terrasar-x precise trajectory estimation and quality assessment. IEEE Trans. Geosci. Remote Sens..

[15] Curlander J.C. (1984). Utilization of spaceborne SAR datafor mapping. IEEE Trans. Geosci. Remote Sens..

[16] Curlander J.C., McDonough R.N. (1991). Synthetic Aperture Radar: Systems and Signal Processing.

[17] Schreier G. (1993). SAR Geocoding: Data and Systems.

[18] Toutin T. (2004). Review article: Geometric processing of remote sensing images: Models, algorithms and methods. Int. J. Remote Sens..

[19] Schwerdt M., Bräutigam B., Bachmann M., Döring B., Schrank D., Gonzalez J.H. (2010). Final terrasar-x calibration results based on novel efficient methods. IEEE Trans. Geosci. Remote Sens..

[20] Srivastava S., Banik B., Le Dantec P., Hawkins R., Murnaghan K. Radarsat-1 Image Quality—A Mission Success. Proceedings of the CEOS SAR Workshop.

[21] Toutin T. (2003). Path processing and block adjustment with radarsat-1 SAR images. IEEE Trans. Geosci. Remote Sens..

[22] Chen P.-H., Dowman I.J. (2001). A weighted least squares solution for space intersection of spaceborne stereo SAR data. IEEE Trans. Geosci. Remote Sens..

[23] Olmsted C. (1993). Alaska SAR Facility Scientific SAR User’s Guide.

[24] Vassilaki D., Stamos A. Interpolating accurate terrasar-x science orbit data. Proceedings of the EARSeL 34th Symposium.

[25] Schubert A., Jehle M., Small D., Meier E. (2012). Mitigation of atmospheric perturbations and solid earth movements in a terrasar-x time-series. J. Geod..

[26] Hong S.H., Sohn H.G., Kim S.P., Jang H.S. (2013). Error budget analysis for geolocation accuracy of high resolution SAR satellite imagery. J. Korean Soc. Surv. Geod. Photogramm. Cartogr..

[27] Schubert A., Jehle M., Small D., Meier E. (2010). Influence of atmospheric path delay on the absolute geolocation accuracy of terrasar-x high-resolution products. IEEE Trans. Geosci. Remote Sens..

[28] Mikhail E.M., Ackermann F.E. (1976). Observations and Least Squares.

[29] Smith A. (2003). Near real-time geocoding of SAR imagery with orbit error removal. Int. J. Remote Sens..

[30] Cote S., Muir S., Srivastava S., Hawkins R. SAR image quality and calibration operations for the radarsat satellites at the canadian space agency. Proceedings of the 2009 International Radar Conference Surveillance for a Safer World (RADAR 2009).

[31] Srivastava S., Banik B., Adamovic M., Gray R., Hawkins R., Lukowski T., Jefferies W. Radarsat-1 image quality—Update. Proceedings of a SAR Workshop.

[32] International R. Radarsat-1 Data Products Specifications (rsi-gs-026). http://gs.mdacorporation.com/includes/documents/R1_PROD_SPEC.pdf.

[33] Shimada M., Isoguchi O., Tadono T., Isono K. (2009). Palsar radiometric and geometric calibration. IEEE Trans. Geosci. Remote Sens..

[34] Eineder M., Breit H., Adam N., Holzner J., Suchandt S., Rabus B. Srtm x-SAR calibration results. Proceedings of the IEEE 2011 International Geoscience and Remote Sensing Symposium (IGARSS’01).

[35] Jehle M., Small D., Meier E., Nüesch D. (2004). Improved knowledge of SAR geometry through atmospheric modelling. Proceedings of the 5th European Conference on Synthetic Aperture Radar EUSAR.

[36] Jehle M., Perler D., Small D., Schubert A., Meier E. (2008). Estimation of atmospheric path delays in terrasar-x data using models vs. Measurements. Sensors.

[37] Hanssen R.F. (2001). Radar Interferometry: Data Interpretation and Error Analysis.

[38] Davies K. (1990). Ionospheric Radio.

[39] Xu Z.-W., Wu J., Wu Z.-S. (2004). A survey of ionospheric effects on space-based radar. Waves Random Med..

[40] Rignot E.J. (2000). Effect of faraday rotation on L-band interferometric and polarimetric synthetic-aperture radar data. IEEE Trans. Geosci. Remote Sens..

[41] Mateus P., Nico G., Tomé R., Catalao J., Miranda P.M. (2013). Experimental study on the atmospheric delay based on GPS, SAR interferometry, and numerical weather model data. IEEE Trans. Geosc. Remote Sens..

[42] Ding X.-L., Li Z.-W., Zhu J.-J., Feng G.-C., Long J.-P. (2008). Atmospheric effects on inSAR measurements and their mitigation. Sensors.

[43] Melchior P. (1974). Earth tides. Geophys. Surv..

[44] Milbert D. Solid Earth Tide. GPS Software Index Page. http://home.comcast.net/~dmilbert/softs/solid.htm.

[45] Penna N.T., Bos M.S., Baker T.F., Scherneck H.-G. (2008). Assessing the accuracy of predicted ocean tide loading displacement values. J. Geod..

[46] Song Y.-S. (2005). Flooded Area Analysis Using Remotely Sensed Data and Gsis in Mountainous Area. Ph.D. Thesis.

[47] Wivell C.E., Steinwand D.R., Kelly G.G., Meyer D.J. (1992). Evaluation of terrain models for the geocoding and terrain correction, of synthetic aperture radar (SAR) images. IEEE Trans. Geosci. Remote Sens..

[48] Song Y.-S., Sohn H.-G., Park C.-H. (2006). An efficient 3-D positioning method from satellite synthetic aperture radar images. Knowledge-Based Intelligent Information and Engineering Systems.

[49] Loew A., Mauser W. (2007). Generation of geometrically and radiometrically terrain corrected SAR image products. Remote Sens. Environ..

[50] Ghilani C.D. (2010). Adjustment Computations: Spatial Data Analysis.

[51] National Geographic Information Institute Production and Dissemination of National Map. http://www.ngii.go.kr/en/contents/contentsView.do?rbsIdx=58.

